# Concrete Made with Iron Ore Tailings as a Fine Aggregate: A Step towards Sustainable Concrete

**DOI:** 10.3390/ma15186236

**Published:** 2022-09-08

**Authors:** Mohamed Moafak Arbili, Muwaffaq Alqurashi, Ali Majdi, Jawad Ahmad, Ahmed Farouk Deifalla

**Affiliations:** 1 Department of Information Technology, Choman Technical Institute, Erbil Polytechnic University, Erbil 44001, Iraq; 2 Civil Engineering Department, College of Engineering, Taif University, P.O. Box 11099, Taif 21944, Saudi Arabia; 3 Department of Building and Construction and Techniques, Al-Mustaqbal University College, Babylon 51001, Iraq; 4 Department of Civil Engineering, Military College of Engineering (NUST), Resulpur 24080, Pakistan; 5 Structural Engineering Department, Faculty of Engineering and Technology, Future University in Egypt, New Cairo 11845, Egypt

**Keywords:** iron ore tailings, sustainable concrete, durability and microstructure analysis

## Abstract

The need for low-cost raw materials is driven by the fact that iron ore tailings, a prevalent kind of hazardous solid waste, have created major environmental issues. Although many studies have focused on using iron ore tailing (IOT) in concrete and have reported positive results, readers may find it difficult to accurately assess the behaviors of IOT in concrete due to the scattered nature of the information. Therefore, a comprehensive assessment of IOT in concrete is necessary. This paper thoroughly reviews the characteristics of concrete that contains IOT such as fresh properties, mechanical properties and durability at different age of curing. The outcome of this review indicates that by using IOT, concrete’s mechanical properties and durability improved, but its flowability decreased. Compressive strength of concrete with 20% substitution of IOT is 14% more than reference concrete. Furthermore, up to 40% substitution of IOT produces concrete that has sufficient flowability and compactability. Scan electronic microscopy results indicate a weak interfacial transition zone (ITZ). The optimum IOT dosage is important since a greater dose may decrease the strength properties and durability owing to a lack of fluidity. Depending on the physical and chemical composition of IOT, the average value of optimum percentages ranges from 30 to 40%. The assessment also recommends areas of unsolved research for future investigations.

## 1. Introduction 

Concrete is a raw material often utilized in construction and serves as the foundation for all infrastructure projects and construction projects worldwide [1,2,3,4,5]. Depending on the kind of concrete and the quantity of cement used, the main components of concrete (cement) have diverse effects on the environment. There are various concerns regarding concrete’s long-term viability since it is used in vast quantities worldwide [3,6]. Environmentalists are quite concerned about the increase in riverbed sand and gravel (mining) used as building materials. Because of the widespread use of concrete and the resulting fast urbanization and industrialization of the global population, there is increased removal of natural sand from riverbeds [7]. Negative effects of sedimentation include an increase in riverbed distance; a drop of the water table; the exposure of bridge substructures; a substantial effect on the river, delta, coastal, and marine ecologies; land failure due to river or coastal erosion; and a reduction in the quantity of deposit source [8]. Additionally, due to restrictions on sand collection from the river, the profitability of the building business has been significantly compromised, leading to a considerable rise in sand costs [9]. Various waste may be used to create fine aggregates for concrete [10,11,12,13]. Much research is focused on improving the quality of environment in different ways [14,15,16,17].

The demand for natural resources has increased along with the continuous rise in living standards, and increasingly more industrial waste is being created [16]. Reusing waste materials as secondary resources is a workable strategy for reducing environmental constraints and achieving sustainability [13]. In this context, millions of tons of industrial byproducts containing harmful substances have previously been absorbed by the concrete industry [18]. One of China’s major industries is the iron and steel sector. Since the implementation of China’s reform and opening-up program thirty years ago, it has advanced quickly. Although it has greatly benefited the economy, it has also caused a significant quantity of industrial waste to be created. Some byproducts, such as slag, which is applied to make cement, have previously been extensively acknowledged for their worth; nevertheless, most dangerous substances, such as iron ore tailings, have been disposed of as garbage. This has caused the environment to seriously deteriorate. Only 7% of China’s 0.6 billion tons of iron ore tailings discharged per year, according to an official statement from 2008, were recycled into new resources [19]. These untreated tailings not only require much space, and damage the air and water supplies, but they also endanger human safety.

Sustainable development is development that meets the needs of the present without compromising the ability of future generations to meet their own needs. The term “sustainable building” describes management that is responsible for formulating a welcoming atmosphere that takes ecological and resources development into consideration [20,21,22]. Owing to its low cost and high performance, concrete is swiftly becoming essential building material in the world [23,24]. However, ecological systems are impacted by the production of cement [25,26]. A considerable source of harmful gas discharges is the manufacture of cement, a key ingredient in concrete [27,28,29]. The world produces 3.6 billion metric tons of material annually [30]. By 2030, the quantity of cement is estimated to exceed 5 billion metric tons [27,31]. Regardless of the fact that each nation’s circumstances are unique, more than half of the world’s OPC produces 11 billion metric tons of concrete annually, with the remaining portions being applied for projects. [32]. Concrete could substitute waste materials for cement to reduce CO_2_ emissions.

**Figure 1:**
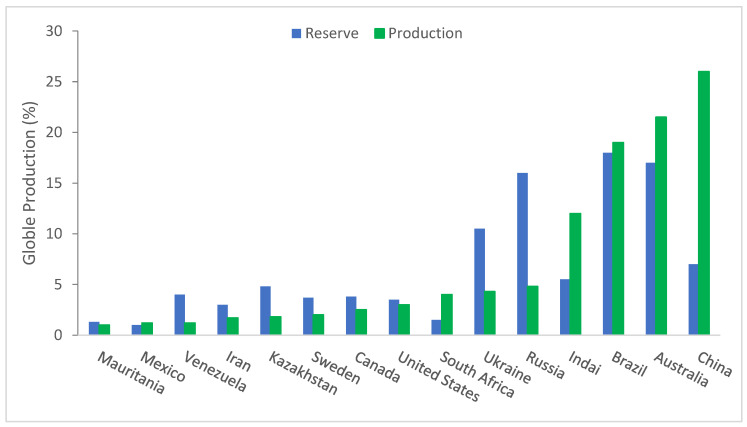
Worldwide production of iron ore (data source [44]).

Owing to worries about potentially hazardous social and environmental effects of waste, the tendency of rising solid waste output has created its environmental disposal a critical problem. Recycling industrial trash is one strategy to reduce these problems [33,34,35]. If recycled resources are correctly assessed before being used as building materials, the construction sector has a significant potential to absorb diverse industrial leftovers. The most widely used metal in the world is iron, which accounts for over 95% of all metals consumed yearly and is a byproduct of iron ore. Worldwide, an average 2 billion metric tons of raw ore is produced yearly. About 9602 million tons of hematite and 3408 million tons of magnetite are the total recoverable resources of iron ore in India. Iron ore usage worldwide is increasing at a 10% rate [36]. The data on iron ore output from 2000 to 2018 are shown in Figure 1. In order to fulfill industrial demand, steel output has expanded recently.

The preparation and performance testing of concrete containing iron mine tailings have made some progress in recent years, owing to efforts by domestic and international academics. Research [37] that used indoor tests to examine the operating efficiency and strength of concrete containing iron ore tailings also conducted a microanalysis of the test data. According to [38], concrete containing various amounts of steel scrap chips has a better gamma-ray shielding effect than regular concrete with only natural sand. Ismail and Al-Hashmi [39] conducted a sequence of experimental tests on the mechanical behavior of concrete made from abandoned iron ore tailings in Iraq, and the findings showed that it performed better than regular concrete and displayed greater compressive strength and flexural strength. Iron ore tailing (IOT) application as fine aggregate in interlocking concrete blocks was examined in the research. Compared to traditional interlocking paver blocks, the physical and mechanical qualities were better [40]. For substitution of 100% natural sand, Liu et al. [41] were able to produce sprayed concrete with a 28-day compressive capacity of 23.4 MPa. In designing cementitious composites, Huang et al. [42] discovered that employing IOT as aggregate produced excellent tensile and compressive capacity. When the IOT replacement of the sand was no more than 40%, Zhao et al. [37] found that the compressive strength was equivalent to that of the reference sample. According to Ma et al. [43], replacing 40% to 60% of the silica sand in autoclaved aerated concrete with IOT might increase the compressive capacity that the C-S-H gel contributes.

**Figure 2:**
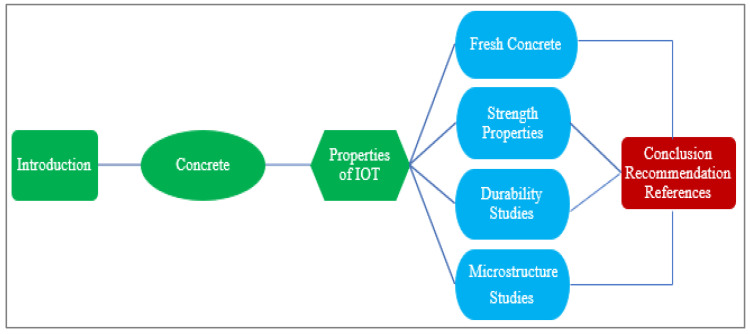
Different sections of the review.

A brief literature review on IOT shows that it has credibility to be used in concrete. Although many researchers focus on the utilization of IOT in concrete and reported a positive response, knowledge is scattered and readers feel difficulty in easily judging the behaviors of IOT in concrete. Therefore, a comprehensive review is required for IOT in concrete. This review provides a detailed study on the physical and chemical properties of concrete containing IOT—when fresh, and its hardening and durability after curing. Microstructure analyses were also reviewed. Finally, the review also identifies research gaps for future studies. Figure 2 shows the different sections of this review.

## 2. Physical and Chemical Properties

**Figure 3:**
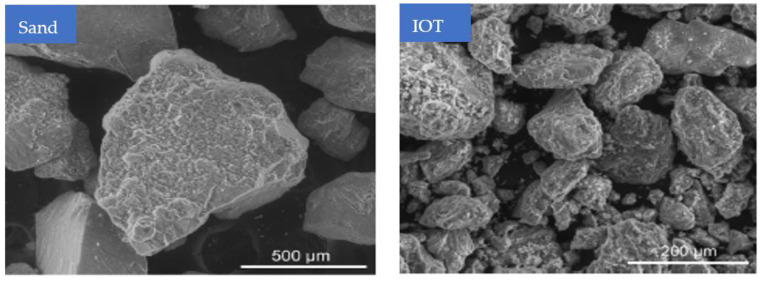
SEM of iron ore particles (used with permission, Elsevier [37]).

Scanning electron microscopy (SEM) images of the two aggregates are shown in Figure 3. The particle shapes of each of them are angular. The surface of the river sand is smoother than that of the tailings, which has a very rough and uneven surface. The surface texture of the concrete decreased its flowability due to its irregularity and roughness, which raised the internal resistance of the components.

**Figure 4:**
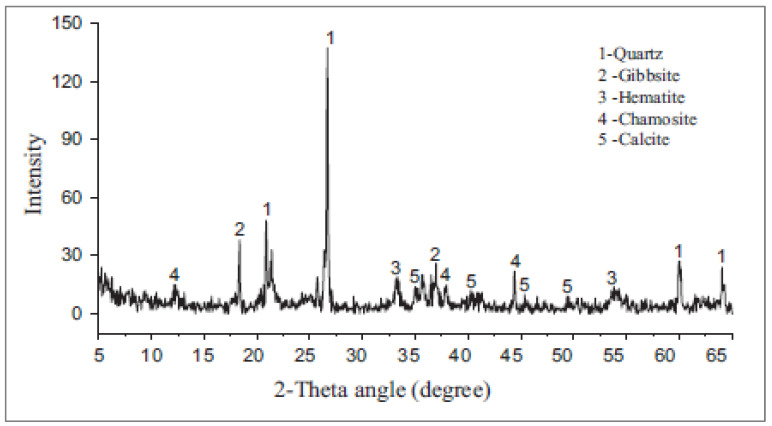
XRD analysis of iron ore (used with permission, Elsevier [48]).

Table 1 depicts the chemical composition of IOT as per past studies. The sum of the chemical composition of IOT from silica, iron, lime, magnesia, and alumina content is greater than 70%. Therefore, IOT has the credibility to be used as a cementitious material. The primary crystalline phases, according to X-ray diffraction (XRD) research, are quartz (SiO_2_), gibbsite, hematite (Fe_2_O_3_), and chamosite [(Fe_2_+Mg)_5_, Al(AlSi_3_O_10_)(OH)_8_], as shown in Figure 4. Owing to the substantial loss of ignition shown in the chemical composition, traces of calcite (CaCO_3_) are also found in the spectrum. According to research, quartz makes up most of the natural sand while other minerals are also present in the tailings [37]. When cement is hydrated, calcium hydrates (CH), which are made of the amorphous silica present in the IOT reaction, develop, producing additional cementitious compounds such as calcium silicate hydrate (CSH) gel. CSH has cementitious qualities that enhance the cement paste’s capacity to bond, enhancing the strength and longevity of the concrete in the process. Additionally, research suggests that CSH fills cement paste spaces, enhancing the strength and durability of concrete [45].

**Table 1:** Chemical composition of iron ore tailings (IOT).

Authors	[37]	[46]	[47]	[48]
SiO_2_	52.06	70.32	72.84	56
Al_2_O_3_	17.14	5.10	4.74	10
Fe_2_O_3_	9.13	10.93	8.88	8.30
MgO	3.68	4.51	6.06	-
CaO	12.75	4.71	5.05	4.30
Na_2_O	0.97	1.30	-	-
K_2_O	0.30	1.14	-	1.50

## 3. Fresh Properties

### 3.1. Slump Flow

**Figure 5:**
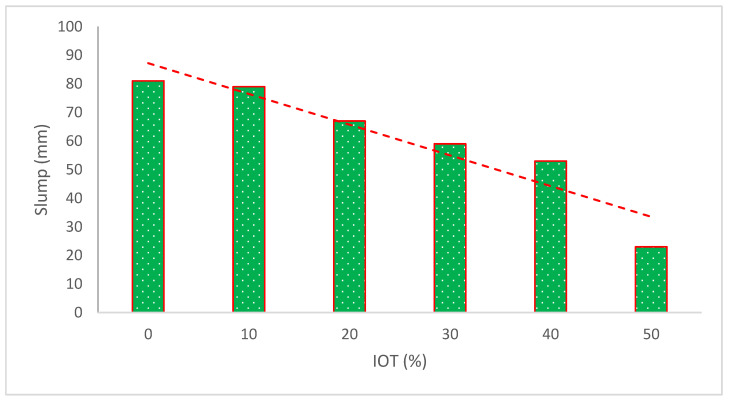
Slump flow results (data source [49]).

In comparison to regular concrete under the same conditions, the slumps and fluidity of concrete mixed with IOT decreased, as indicated in Figure 5 and Table 2. This effect occurs as a result of the roughness and coarse aggregate occlusion of the iron ore tailing particles, which greatly enhances cohesion in the tailing mixes. Additionally, compared to natural sand, iron ore tailings absorb more water. As a result, the tailing mixtures have poor liquidity. The loss of slumps exhibited a linear relationship with the rise in the amount of IOT, per the experiment on the holding value of slumps for 1 h. Concrete containing iron tailing sand had enhanced performance in agglomeration, as shown by the 12% loss value of concrete with 45% for 1 h. Owing to the tailing mixes’ poor solubility, cement paste and aggregate quickly separated in the experiment. Segregation and a decrease in water retention capacity would result from improper mix proportions. The slumps are all more than 150 mm in all circumstances, making them practicable. In conclusion, when the quantity is lower than 45%, iron tailing sand might assure workability in shipping and pouring [46].

According to research, the first slump value improved as IOT content grew, especially when IOT substitution was less than 30% and water content remained constant. The combination that included 30% IOT had the largest drop, measuring 195 mm, together with good cohesion and water retention. The reason for this was that the finer IOT made the mortar more fluid, which reduced the coarse aggregate’s resistance to motion. However, when the IOT content increased and reached a level of 30% or more, the slump value began to decline. This was done to avoid the concrete mixture becoming too thick if there was too much IOT in the finer-size powder [50]. The IOT has larger particles than cement; less water is needed due to the smaller specific surface area. The IOT also has a crystalline structure and a reduced water absorption rate [43]. However, research revealed that the workability of concrete was reduced when river sand was substituted for IOT. When IOT was increased in the concrete mix to 100%, a slump reduction of 38% was seen in comparison to the control. In comparison to IOT containing concrete, freshly mixed traditional concrete was more cohesive and flowable. This could be explained by IOT’s greater water absorption capacity, angular surface area, and fine texture.

The fineness modulus for IOT is 1.05, which is less than half the minimum 2.3 value required by the ASTM C33 standard for sand [51], reducing the flowability by increasing the water demand in the concrete. The fineness modulus of the material applied to make concrete directly relates to the concrete’s workability [52]. According to one study [53], the improvement in the slump workability of concrete was seen with IOT. The findings indicate that as iron waste increased, the slump progressively and marginally lessened. This indicates that concrete has excellent durability and is workable within a reasonable range [54]. As tailings content increases, the slump becomes smaller. Because of its rough surface and large specific surface area, the iron ore tailings have a strong affinity for water [37].

**Table 2:** Summary of slump flow.

Reference	Iron Ore Tailing (IOT)	Slump (mm)	Remarks
[46]	0%, 25%, 35%, and 45%	195, 180, 175, and 170	Decreased
[36]	0%, 10%, 20%, 30%, 40%, and 50%	45, 45, 45, 46, and 32	No effect up 40% but 50% substitution caused decrease
[47]	0%, 20%, 40%, 60%, 80%, and 100%	220, 210, 190, 185, 170, and 160	Decreased
[49]	0%, 10%, 20%, 30%, 40%, and 50%	67, 55, 38, 25, 25, and 23	Decreased
[55]	0%, 10%, 20%, 30%, and 40%	750, 758, 772, 783, and 791	Increased
[56]	0%, 10%, 20%, and 30%	127, 140, 152, and 200	Increased
[54]	0%, 6%, 12%, 18%, 24%, and 30%	65, 63, 60, 58, 57, and 55	Decreased
[57]	0%, 10%, 20%, 30%, and 40%	81, 79, 67, 59, and 53	Decreased
[41]	0%, 20%, 40%, 60%, 80%, and 100%	70, 130, 115, 70, 95, and 70	Increased up to 80% substation
[58]	0%, 5%, 7%, 10%, 13%, 15%, 17%, 20%, 25%, and 30%	100, 85, 70, 64, 56, 55, 48, 44, 30, and 25	Decreased
[50]	0%, 10%, 20%, 30%, 40%, 50%, and 60%	50, 55, 55, 60, 60, 65, and 75	Increased

### 3.2. Bleeding

**Figure 6:**
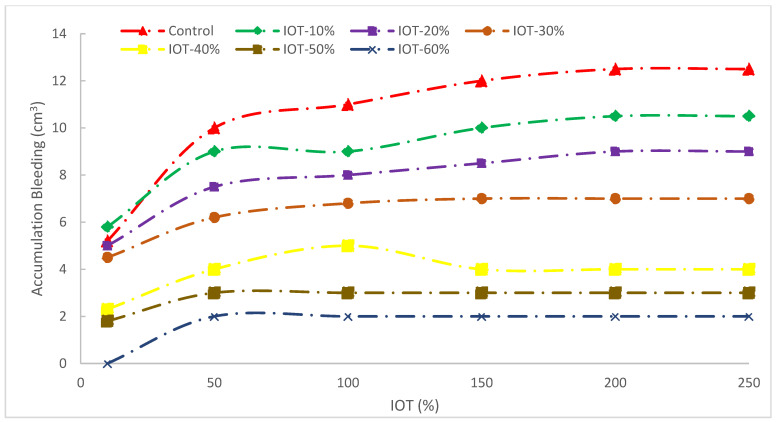
Bleeding of concrete with IOT [50].

Figure 6 displays the quantity of concrete seeping over time with various IOT quantities. The outcomes of the bleeding experiments demonstrated that the inclusion of IOT has a good effect by reducing both the capacity and rate of bleeding. 

For instance, the bleeding capacity at 30 min was lowered by 52% and the bleeding rate by 48% for the mix containing 30% IOT. The bleeding tests also revealed that when the IOT content was lower than 30%, the bleeding rate increased after 30 min. The finer size of IOT with a particular surface, which decreased the quantity of free water in the mixture compared to natural river sand, should be blamed for the decrease in bleeding rate and bleeding capacity [50].

### 3.3. Compacting Factor

**Figure 7:**
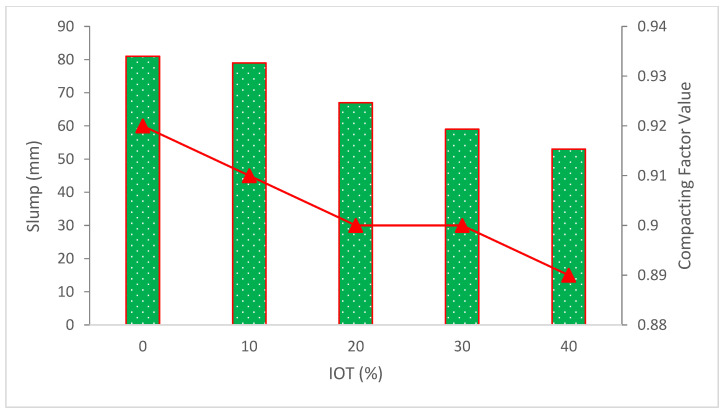
Compacting factor results (data source [57]).

The results of the compacting factor test vary from 0.89 to 0.92, as shown in Figure 7. The compacting factor value for normal weight concrete varies from 0.8 to 0.92 [14]. With a rise in tailings content, the compacting factor declines. This may be due to iron ore tailings’ fineness, which allows the particles to fill more voids in new concrete and produce a heavier compacted mass [57]. However, it should be emphasized that all of the mixtures have high compatibility, with compacting factors ranging from 0.8 to 0.92. Similar research asserts that when IOT is substituted, the compacting factor value decreases [49].

## 4. Mechanical Strength

### 4.1. Compressive Strength (CS)

**Figure 8:**
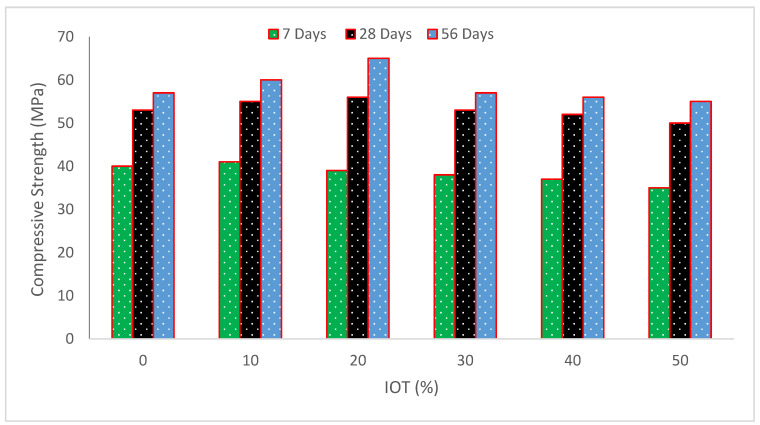
Compressive strength of concrete (data source [36]).

Figure 8 and Table 3 depict concrete’s compressive strength (CS) at different IOT percentages. It was discovered that the material’s workability and CS were dramatically reduced when natural aggregate was completely replaced with tailings. For specimens that were steam-cured for two days, the CS of the tailing mixes decreased by less than 11% while the flexural strengths increased by up to 8% in comparison to the control mix. However, when the replacement level was no more than 40%, for 90 days of standard cured specimens, the mechanical behavior of the tailing mixes was comparable to that of the control mix [37]. The 28-day CS is greater at the 40% replacement level than the reference mix and other replacement percentage blends [49]. At all ages, concrete containing 25% IOT consistently outperformed reference concrete in terms of CS [48]. According to research reported in [59], IOT powder lowered CS, but with increased curing time, the strength was comparable to that of normal cement paste. 

In contrast to 28-day conventional cured counterparts, the heat-treated specimens had greater CS. This may be accounted for by the acceleration of the hydration process during high-temperature curing, which results in a notable increase in strength at a young age. The quick hydration products that form around the unhydrated cement grains, on the other hand, impede further hydration and increase porosity, which has a detrimental effect on the strength development [60]. The CS of the tailing mixes after 28 days decreased and then increased. In particular, the 28-day compressive strength performance was better and the long-term CS was marginally greater than that of the control mix when the proportion of natural aggregate replaced by tailings in the concrete was 35% [46]. The greater substitution ratio of iron ore tailings increased the number of dangerous voids in the concrete mix, and the CS of the concrete with a 45% substitution of natural aggregate by tailings was unsatisfactory. When mixing is inadequate, the concrete is more prone to the layered bleeding phenomena, the paste and aggregate’s binding strength develops unevenly, and the concrete’s total CS declines [61].

**Table 3:** Summary of compressive strength.

Reference	Iron Ore Tailing (IOT)	Compression Strength (MPa)
[37]	0%, 20%, 40%, 60%, 80%, and 100%	7 Days
		78, 85, 80, 75, 72, and 70
		28 Days
		95, 92, 87, 85, 83, and 81
[46]	0%, 25%, 35%, and 45%	3 Days
		16, 15, 17, and 14
		7 Days
		28, 27, 30, and 25
		28 Days
		39, 38, 40, and 35
[36]	0%, 10%, 20%, 30%, 40%, and 50%	7 Days
		40, 41, 39, 38, 37, and 35
		28 Days
		53, 55, 56, 53, 52, and 50
		56 Days
		57, 60, 65, 57, 56, and 55
[62]	Replacement with cement 0%, 0.4%, 0.8%, and 1.2%	75, 58, 50, and 53
[40]	0%, 10%, 20%, and 80%	7 Days
		57.6, 55.6, 43.4, and 43
		70 Days
		62.6, 62, 51, and 48.9
[47]	0%, 20%, 40%, 60%, 80%, and 100%	7 Days
		88, 92, 95, 90, 87, and 83
		28 Days
		105, 112, 120, 111, 104, and 95
[48]	0%, 25%, 50%, 75%, and 100%	7 Days
		32, 31.9, 30.2, 32.8, and 29.3
		14 Days
		34.3, 35.7, 35.3, 35.1, and 33.1
		28 Days
		38, 42.9, 42, 41.9, and 38.5
[50]	0%, 10%, 20%, 30%, 40%, 50%, and 60%	7 Days
		23.7, 25.1, 26.9, 28.9, 29.8, and 28.7
		28 Days
		40.8, 40.1, 41.5, 42.8, 40.1, 38.6, and 35.8
		90 Days
		41.2, 41.9, 42.6, 43.1, 39.8, 37.8, and 35.1
[49]	0%, 10%, 20%, 30%, 40% and 50%	3 Days
		23.83, 23.03, 21.65, 24.27, 26.08, and 25.94
		7 Days
		27.17, 32.92, 34.15, 35.02, 32.48, and 38.91
		28 Days
		38.58, 49.28, 50.27, 21.59, 55.10, and 53.76
		56 Days
		41.05, 50.22, 53.13, 55.13, 55.13, 56.59, and 54.10
[63]	0%, 10%, 20%, 30%, 40%, and 50%	7 Days
		37, 30, 33, 33, 30, 28, and 25
		28 Days
		46, 46, 47, 50, 45, 44, and 43
[55]	0%, 10%, 20%, 30%, and 40%	7 Days
		25, 28, 32, 31, and 26
		28 Days
		32, 32, 38, 33, and 35
[56]	0%, 10%, 20%, and 30%	7 Days
		95, 95, 85, and 88
		28 Days
		120, 142, 122, and 119
[54]	0%, 6%, 12%, 18%, 24%, and 30%	14 Days
		35, 36, 39, 23, 21, and 20.
		28 Days
		39, 40, 41, 35, 28, and 25
[57]	0%, 10%, 20%, 30%, and 40%	14 Days
		25, 33, 33, 37, and 28
		28 Days
		30, 38, 39, 42, and 35
[41]	0%, 20%, 40%, 60%, 80%, and 100%	1 Days
		5.68, 9.35, 5.51, 4.67, 4.43, and 2.53
		3 Days
		24.51, 27.57, 20.70, 18.78, 15.52, and 12.53
		7 Days
		32.28, 36.69, 31.69, 29.38, 24.04, and 20.49
		28 Days
		43.09, 45.17, 40.44, 35.09, 32.23, and 23.39
[58]	0%, 5%, 7%, 10%, 13%, 15%, 17%, 20%, 25%, and 30%	41.37, 37.48, 38.82, 38.75, 39.18, 40.07, 44.64, 40.76, and 40.20
[64]	Fly Ash	43, 46, 49, and 42
	100%, 90%, 80%, 70%	
	IOT	
	0%, 10%, 20%, and 30%	

It should be highlighted that concrete made with 12% iron waste had a 15% higher CS than regular concrete after 28 days. The mix ratio was primarily intended to provide 33 MPa at 28 days, but more may be achieved at 7 days by adding 12% iron waste, which is equivalent to 35 MPa. Therefore, using 12% iron waste in concrete may be advised if the concrete has to attain its maximum CS in the shortest amount of time [54]. The CS steadily rises when manufactured sand is substituted with 0%, 20%, and 40% IOT. The samples with the greatest CS are the concrete mortars that contain 40% IOT. Additionally, at 7 and 28 days, when the tailings concentration is no higher than 80%, the concrete mortars’ CS is equivalent to that of the control [47]. All of the suggested mixes achieved a CS of more than 35 MPa in terms of mechanical strength, with up to 80% of natural aggregates replaced by IOT. It implies that they are appropriate for roads used by commercial vehicles. Mixtures containing 10 and 20% IOT had CS that was more than the minimum acceptable limit of 50 MPa. As a result, they may be used on roads for special vehicles or subjected to severe abrasion [40].

**Figure 9:**
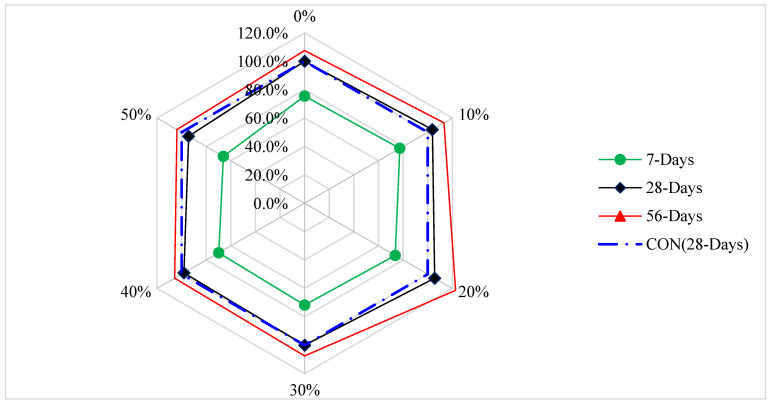
Strength–age relation of CS (data source [36]).

Figure 9 shows the strength–age relation of CS with varying substitution ratios of IOT at 7, 28, and 56 days of curing. The CS of control concrete at 28 days is taken as reference strength. Maximum CS was noted at 20% replacement of IOT with sand. At 7 days of curing, CS is 37% less than reference CS at the 20% substitution ratio of IOT. At 28 and 56 days, CS at 20% substitution of IOT with sand is 6 and 26% higher than reference concrete. It can be concluded that IOT improved CS considerably as the curing days increased.

### 4.2. Split Tensile Strength (TS)

**Figure 10:**
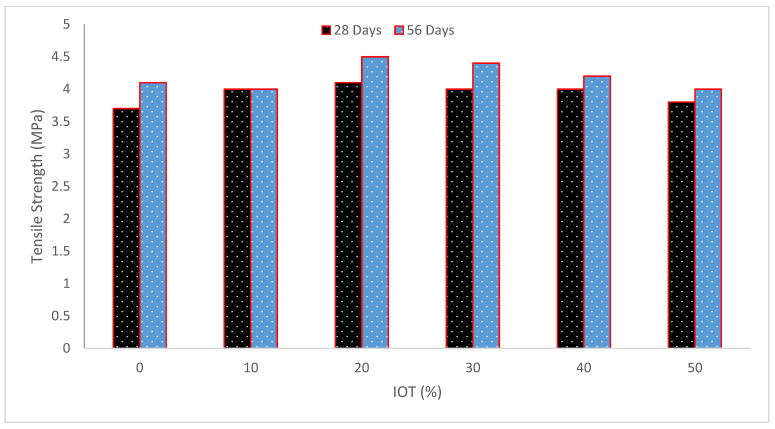
Tensile strength (data source [36]).

Figure 10 and Table 4 depict concrete’s tensile strength (TS) at different IOT percentages. The findings showed that concrete specimens containing 25% IOT significantly enhanced the TS. Even though the TS fell after 7 and 28 days of curing with an increase in IOT, it always remained greater than the control concrete. The splitting TS of IOT concrete mixes did, however, significantly improve between the findings at 7 and 28 days [48]. One study found that using IOT as aggregate obtained good TS and CS in engineered cementitious composites [42]. Concrete TS at 28 days was 20.5%, 12.8%, 7.7%, and 2.5% for substitutions of 25, 50, 75, and 100% of IOT, respectively. These exceeded the control combination in number. According to observations by Gama and Ejeh [65], adding 20% IOT to concrete would increase its TS by 22.8% above the control mix, closely matching the 25% substituted sample. The stronger bonding between aggregates and cement paste created by the smaller tailing particles may be the cause of the increased TS shown in IOT concrete [66]. Research also found that the inclusion of the tailings somewhat increased the splitting TS and flexural strength (FS) values [57]. It was discovered that when the iron filing was added to the concrete mix, the CS of the concrete progressively rose, but the TS changed only slightly when the iron filing ratio was raised to more than 10% [67]. By raising the iron waste ratio to 12%, the CS and TS were somewhat enhanced [54]. When compared to reference concrete, the TS shown by the concrete with iron ore tailings aggregate improved by 4.8% with age [68]. However, because of the greater fines content, the concrete with iron ore tailings aggregate would need more water to mix, which weakens the link between the aggregate and cement paste, and lowers the TS [69].

### 4.3. Flexural Strength (FS)

**Figure 11:**
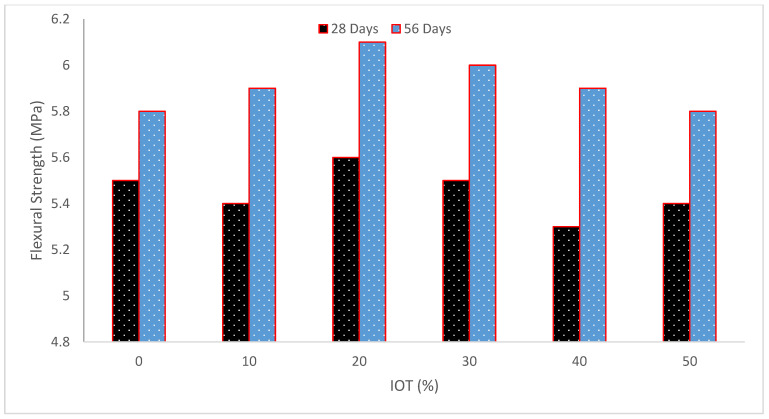
Flexural strength (data source [36]).

Figure 11 and Table 5 depict concrete’s flexural strength (FS) at different IOT percentages. Flexural capacity decreases when iron waste is increased by more than 12%. As a result, it can be claimed that using iron waste in restricted quantities to substitute sand would enhance the strength of concrete. In light of this, it may be said that recycling materials improve concrete’s structural qualities while also helping to preserve the environment [54]. Early tests of the FS after the tailings were added to the mixture revealed no deterioration. At 3 and 7 days, T20, T30, and T100 even outperformed T0 in terms of FS.

At 28 days, however, all the tailing mortars had a strength reduction of 10 to 17% whereas the control mix still had an FS of 18.6 MPa. The flexural strength of T0 reached 22.5 MPa after 90 days, and the strength difference between T0 and the tailing mortars widened even more. When the tailings concentration was between 20 and 50%, the FS decreased by around 20% and by 29% for T100. The control mix’s FS was equivalent to that of its 28-day standard cured counterpart for steam curing, while all of the tailing mortars had strengths that were greater than those of their 28-day standard cured counterparts and were comparable to the control mix [37]. The standard mix outperformed IOT replacement mixes in terms of maximal FS [49]. The research found that the inclusion of the tailings somewhat increased the value of flexural strength [57]. Because it achieves maximum strength in the shortest amount of time, iron waste at a percentage of 12% is found to be more effective than other percentages in both CS and FS, and it appears that adding iron waste to concrete at a rate higher than 12% would reduce its strength [54]. When IOT and river sand are combined at a rate of 20% IOT and 80% river sand, the test result demonstrates that the 28-day compressive strength, indirect tensile, and FS values are equivalent to the control mix [48].

## 5. Durability

### 5.1. Water Absorption

**Figure 12:**
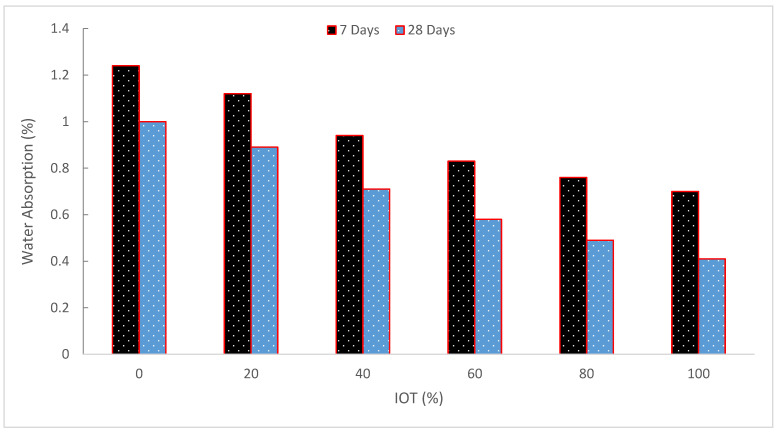
Water absorption (data source [47]).

Figure 12 shows the impact of IOT concentration on the water absorption of UHPC specimens at 7 and 28 days. Evidently, compared to T0, all IOT samples absorb less water. For both curing ages, a reduction in water absorption is shown when the IOT replacement is increased by 9.7%, 24.2%, 33.1%, 38.7%, and 43.5%. At 28 days, the water absorption shows a similar downward pattern. 

The previous findings demonstrate that water absorption decreases as IOT concentration increases, making UHPC mortars very impermeable. The mortars’ macropores and micropores fill with fine IOT particles as the hydration products rise [70]. Therefore, IOT have a favorable impact on impermeability. Low water absorption was also discovered by Chan et al. [71] to be advantageous for concrete durability. Because so much cement was used to create UHPC, many of the cement particles did not participate in the hydration. This caused a gap between the nonhydrated cement particles and the hydration products, which was bad for strength growth. However, in the case of the water absorption test, the reduction in water absorption is mostly caused by the interior structure’s progressive densification. There is an increase in hydration products with the passage of time. In addition, the UHPC’s interior pores are being filled by the replenishment of iron ore tailings. According to [48], the control specimen hardly changed when the cure time increased and the water absorption decreased. The increased hydration process and fineness of the IOT, which occupied both the macropores and micropores in the mix, are likely to blame for the relative reduction in absorption capabilities. In other words, the IOT component had an impact on the concrete’s pore and grain refinements [70]. When ceramic waste was employed as coarse aggregate, Correia et al. [72] found a similar tendency in related studies. They claimed that when the percentage of ceramic particles in the concrete mix increases, water absorption via immersion increases. However, according to Neville [73], none of the test results for water absorption exceeded 10% by mass. Chan and Sun [71] claim that more water adsorption has a detrimental effect on the workability and durability of concrete.

### 5.2. Chloride Ion—Penetration Resistance

**Figure 13:**
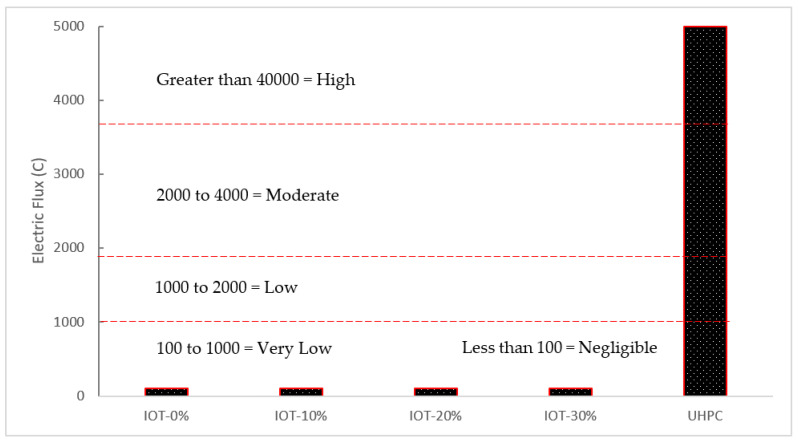
Chloride resistance (data source [56]).

The Coulomb electric flux test, which is used for the chloride permeability rating, is used to assess the impact of IOT on the planned UHPC’s chloride resistance. The findings are displayed in Figure 13. When IOT is added in amounts no more than 20%, it is evident that the specified ultrahigh performance concrete (UHPC) combinations exhibit good chloride resistance. When IOT dose reaches 30%, however, the planned HPC mixes’ chloride resistance is drastically reduced. Figure 13 shows that the IOT10 and IOT20 combinations had total charges of 85 and 92 °C, respectively, which are 4.1% and 13.6% greater than the reference mixture (81 °C). The overall charge rises to 107 °C with the additional inclusion of IOT (IOT30), a 32.1% increase over IOT0. It should be ascribed to the low IOT activity, which caused the hydration products to drop and the microstructure of the intended UHPC to deteriorate. UHPC’s durability suffers as a consequence. However, it should be noted that practically all constructed UHPC, including IOT, may have their chloride permeability classified as negligible, which has significant benefits over regular strength concrete. Considering its resistance to chloride ion penetration, IOT can be used in UHPC.

### 5.3. Shrinkage

**Figure 14:**
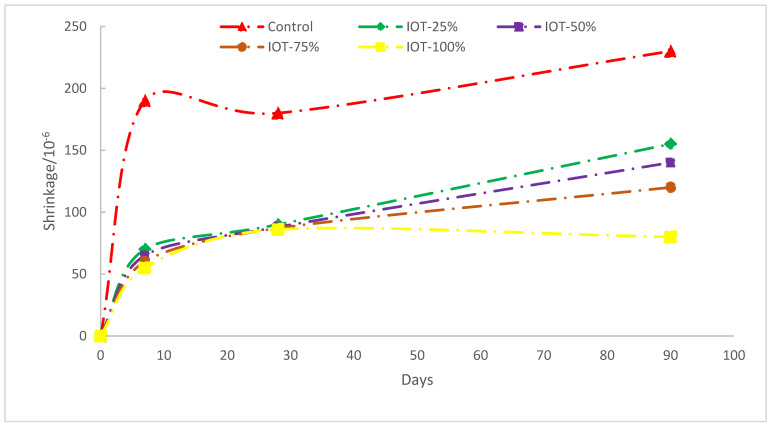
Drying shrinkage (data source [48]).

In general, shrinkage is considered as a significant durability characteristic of hardened concrete. Concrete durability may be reduced if harmful contaminants enter via fractures caused by dry shrinkage [17,74]. The effects of drying shrinkage are seen in Figure 14. When compared to concrete manufactured with river sand, it was found that IOT concretes dried with less shrinkage. The shrinkage of the control concrete specimen was greater and diverged from IOT concrete during the course of the test. Ninety-day concrete shrinkage values for T25, T50, T75, and T100 were 24%, 43%, 46%, and 58% less than control specimens, respectively. The smaller particles that filled the concrete’s micropores and improved the pore structure were thought to be responsible for IOT decreased drying shrinkage rates. This conclusion is in complete agreement with Zhou et al. [75]. Additionally, research revealed that concrete substitution dry shrinkage caused by microfilming gaps in mineral materials was also detected [34]. The porous nature of IOT, which would collect more water and release moisture slowly during the drying phase of concrete, similar to the situation of furnace bottom ash, might be another explanation for IOT’s reduced drying shrinkage [76]. Feng et al. [77] noted drying shrinkage in an iron mine tailing sample that was lower than that of the control samples. They indicated that concrete of grades C30 and C60 dried with less shrinkage than the control specimen. Additionally, Zhang et al. [78] examined the characteristics of concrete made using manufactured sand and iron ore tailings as fine aggregate. They claimed that mixed sand shrinks less while drying than river sand concrete, and that this shrinkage lessens as IOT increases. When furnace bottom ash (FBA) was utilized as fine aggregate, Bai et al. discovered a similar tendency in a comparable investigation. With an increase in FBA, drying shrinkage at a constant water-to-cement ratio decreased [79]. Yan and Jianyong [80] investigated the shrinkage and creep of high-performance concrete. They claimed that by filling the pores and voids that are damaging to the microperformance of the concrete, ultrafine-ground granulated blast furnace slag and silica fume added to concrete decreased creep and shrinkage.

## 6. Microstructure (Scanning Electron Microscopy)

**Figure 15:**
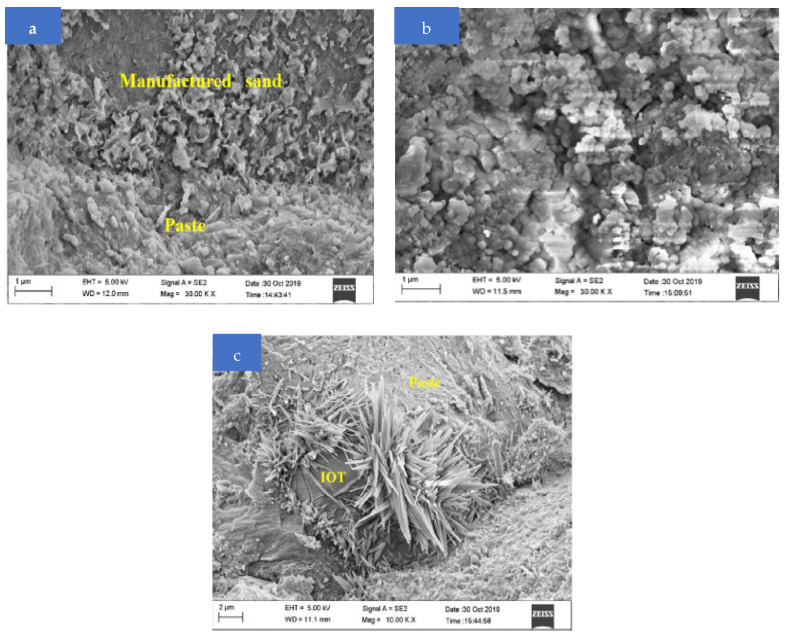
SEM results: (**a**) control; (**b**) 40% IOT; (**c**) 100% IOT (used with permission, Elsevier [47]).

The ITZ between the paste and MS seems to be extremely thick as the curing age increased, there are hardly any noticeable flaws, and the connection between them looks to be very strong at 28 days, as shown in Figure 15a,b. Utilizing silica fume, the ITZ’s packing is densified [81].

T40 has the maximum compressive strength at age 28. As shown in Figure 15b, the ITZ of the synthetic sand, paste, and IOT is relatively thick, and the C-S-H gels are generally homogenous. The microstructure is made more homogeneous by the filling effect of IOT and the pozzolanic reaction of some of the finer IOT. Owing to the tiny, higher surface energy particles, the structure of concrete mortars becomes denser and more homogeneous. T40 has exceptional compressive strength as a result. The morphology of T100 is seen at low magnification in Figure 15c. This image shows a significant number of air spaces that are trapped. In addition, a significant quantity of AFt is shown in the ITZ between the IOT and paste in Figure 15c. Under stress, AFt splits and breaks quite readily. A heterogeneous and uneven microstructure is a result of both AFt and the trapped air spaces. Thus, among all specimens at all curing ages, T100 had the lowest compressive strength. Close packing density, particularly at low w/c, has a direct impact on microstructure development [82]. The interfacial zone and the bulk paste of the UHPC had comparable micromechanical characteristics, according to earlier nanoindentation research [83]. The major cause of this is the ITZ’s usage of silica fume, which densifies the packing [81]. Furthermore, the high water-retention capacity and high homogeneity of the fresh UHPC are associated with the dense ITZ; without these properties, bleeding water may collect around the aggregate and result in a porous and fragile ITZ. The IOT size is between that of binding materials and sand, which may also describe why the replacement of 40% IOT displays the optimum macroscopic performance. When providing IOT at suitable ratio, these materials may provide a satisfactory dense concrete by fillings the voids in aggregates.

## 7. Thermal Stability

**Figure 16:**
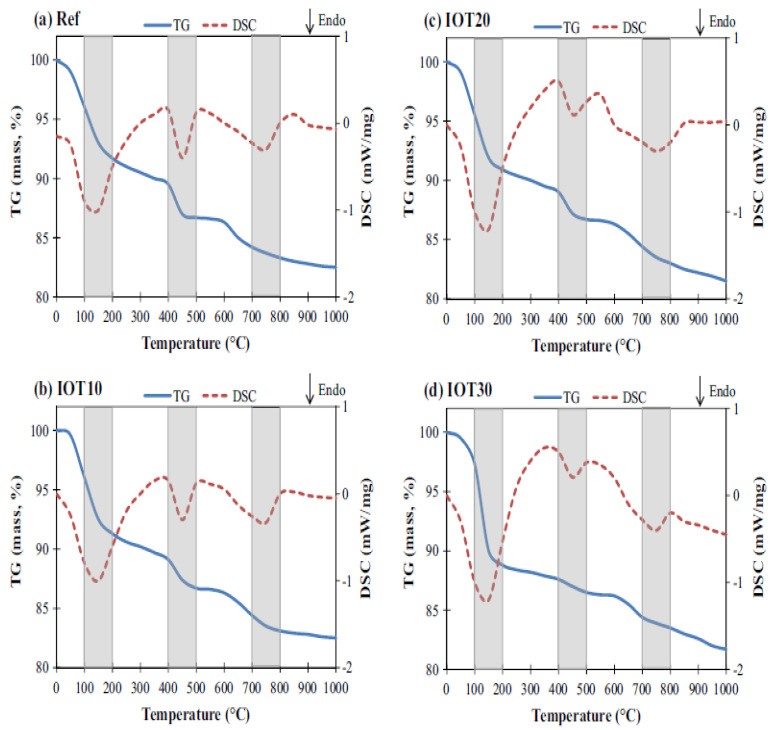
Thermal analysis curves [64].

Figure 16 depicts the samples’ thermogravimetry (TG) and differential scanning calorimetry (DSC) curves. By observing the weight change during specimen heating, TG-DSC was utilized to calculate the thermal stability of geopolymer and its proportion of volatile components.

The weight loss in the references IOT10, IOT20, and IOT30 was attributed to the breakdown of portlandite (Ca(OH)_2_) and calcium carbonate (CaCO_3_) into CaO and CO_2_, respectively, and the evaporation of water (from liquid activator and C-S-H) and water at various temperatures [84]. According to earlier written works [85], clear endothermic peaks were discovered around these temperatures. It is consistent with dehydration of Ca(OH)_2_(CH), which occurred between 447 and 462 °C. Wei et al. [86] discovered another similarity. They used TG-DSC to measure the CH concentration at these temperatures. Importantly, the TG analyses were used in all of these investigations to estimate the calcium hydrate (CH) quantities based on the mass loss. Because there is less mass loss at 440 °C compared to the control sample, the findings indicated in Figure 16a–d show that the amount of calcium hydrate (CH) decreases with an increasing substitution ratio of IOT. The Ca(OH)_2_ is decomposed by the addition of IOT, resulting in the creation of C-S-H. However, even after the addition of 30% IOT, an endothermic peak that corresponds to the breakdown of Ca(OH)_2_ can still be seen, proving that not all of the Ca is transformed into C-S-H. By consuming Ca(OH)_2_, the addition of IOT causes the matrix to produce more C-S-H. Calculating the mass loss at 440 °C, which is caused by the breakdown of Ca(OH)_2_, may be used to determine the quantity of Ca(OH)_2_. Ca(OH)_2_ amounts are shown in Figure 16. With the addition of IOT, it can be observed that the Ca(OH)_2_ contents decrease, indicating its conversion to C-S-H. The findings of the rising compressive strength are further supported by the lowered Ca(OH)_2_ and the resulting creation of more C-S-H. The weight loss at about 120 °C caused by the evaporation of water (calcium silicate hydrate gel) rises with the concentration of IOT, indicating that calcium silicate hydrate gel is being produced as a result of the addition of IOT. The IOT’s role in the development of calcium silicate hydrate gel is demonstrated by the reduced Ca(OH)_2_ and the resulting formation of additional C-S-H.

## 8. Cost and Environmental Evaluation

**Figure 17:**
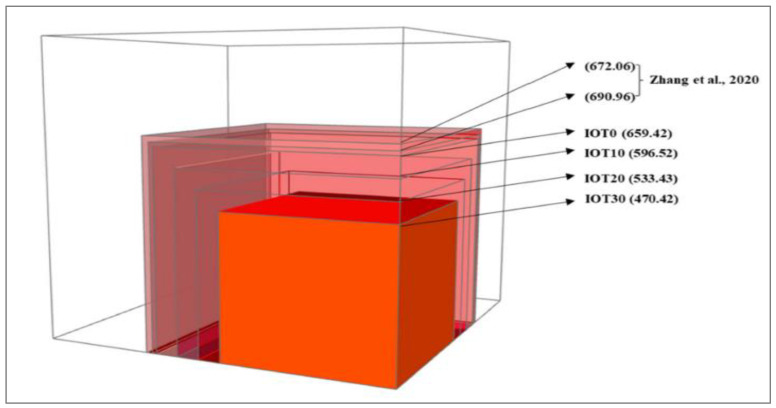
Emission of carbon dioxide [56].

Owing to its superior mechanical qualities compared to conventional concrete, UHPC may be considered an environmentally benign material. As a result, it is possible to create lightweight concrete structures while using less raw resources overall. Additionally, because of its great resilience, concrete may have a longer service life and lower cost maintenance in the future. In addition to the benefits already described, the emission CO_2_ index of the planned UHPC with IOT has to be assessed since IOT replaces some of the cement. A carbon footprint is used to evaluate how sustainable a constructed UHPC is, and Figure 17 shows the estimated CO_2_ emission (kg/m^3^). It is evident that the installation of IOT reduces CO_2_ emissions. For instance, a typical UHPC mixture without IOT emits 690.96 kg/m^3^ of CO_2_ [47].

When IOT is added at a 30% concentration, this number is dramatically lowered to 470.42 kg. IOT has also been used to substitute for river sand as the fine aggregate. However, according to the reference, even with 100% replacement, the CO_2_ emission is still 672.06 kg/m^3^ [47], considering the manufacture of OPC is the primary supply of CO_2_ discharges for the UHPC mixture. Additionally, the application of IOT in UHPC may lessen the burden on landfills brought on by IOT. In general, IOT may be utilized to create an advanced, ecofriendly UHPC. Therefore, from the standpoint of an environmentally responsible UHPC design, it makes sense to employ IOT as the cementitious material.

Research reported in [87] found that incorporating the use of mine tailings into mining firms’ operations may lower rehabilitation costs, lessen the impact of mine closures on mining towns, and stimulate new financial interest. Similar research, which determined that 70–80% of concrete is made up of aggregates, enormous quantities of iron ore tailings from mines in Western Australia might be used to make aggregates for concrete. Because there may be a significant reduction in the amount of IOT, their usage could have a favorable impact on the environment. Additionally, this may result in less harmful ecological consequences and increase the condition of sustainability in business operations of iron ore mining. The expense of maintaining and monitoring mine tailings, on the other hand, is crucial to the longevity of any mine. This results from strict laws and rules that the relevant government agencies have set in place for mining corporations to follow while handling their tailings. Mining businesses do this by allocating a dedicated cost for this reason, which is often involved in their expense of manufacture. In this case, thorough usage of mine tailings will provide additional revenue to help mining businesses cover this expense once the mine is closed [68].

## 9. Conclusions

The performance of concrete with incorporated IOT is reviewed for several studies carried out during the last ten years. More than 80 papers were critically examined and summarized in this work. The following conclusions may be made in light of this review:

- Physical properties show that IOT particles are rough and angular, which reduce the flowability of concrete.
- The chemical composition and XRD analysis of IOT show that IOT has the ability to be utilized as a binding material.
- Fresh properties of concrete such as slump flow and compactability decrease with added IOT. However, up to 40% substitution of IOT shows that concrete has sufficient flowability and compactability. Furthermore, for higher doses, a plasticizer is recommended.
- To certain limit, mechanical strength improves with IOT substitution due to micro filling voids and pozzolanic reaction. Different researchers recommend different optimum doses. However, the typical optimum dose varies from 30 to 40%. However, a higher dose of IOT (greater 40%) adversely affects the mechanical strength of concrete.
- Compressive strength of concrete with 20% substituted IOT is 14% more than reference concrete.
- Water absorption, chloride ion penetration, and dry shrinkage decrease considerably with the substitution of IOT.
- SEM results indicate that ITZ (cracks) improved with the substitution IOT due to filling voids and pozzolanic reaction.

Finally, the overall studies recommend that up to 40% IOT has the credibility to be used in concrete without any harmful effect on strength and durability properties.

## 10. Recommendations 

Although IOT enhanced concrete strength and durability properties, its low tension capacity results in sudden collapse without any indication. Therefore, more study is suggested to enhance the tensile strength of dune-based sand with the addition of fibers. Additionally, as IOT adversely affects the flowability of concrete, further research is recommended to apply a treatment (heat and alkaline solution). A detailed investigation of IOT as fine aggregate at elevated temperatures should be conducted.

## Figures and Tables

**Table 4:** Summary of tensile strength.

Reference	Iron Ore Tailing (IOT)	Tensile Strength (MPa)
[36]	0%, 10%, 20%, 30%, 40%, and 50%	28 Days
		3.7, 4.0, 4.1, 4.0, 4.0, 4.0, and 3.8
		56 Days
		4.1, 4.0, 4.5, 4.4, 4.2, and 4.0
[62]	Replacement with Cement 0%, 0.4%, 0.8%, and 1.2%	6.1, 6.0, 5.3, and 5.5
[48]	0%, 25%, 50%, 75%, and 100%	7 Days
		3.1, 3.9, 3.8, 3.6, and 3.4
		28 Days
		3.9, 4.7, 4.4, 4.2, and 4.0
[55]	0%, 10%, 20%, 30%, and 40%	28 Days
		3.5, 3.2, 3.5, 3.1, and 3.3
[57]	0%, 10%, 20%, 30%, and 40%	28 Days
		3.54, 3.72, 3.75, 3.91, and 4.0
[58]	0%, 5%, 7%, 10%, 13%, 15%, 17%, 20%, 25%, and 30%	3.06, 2.34, 2.43, 2.67, 2.92, 2.72, 2.64, 2.75, 2.46, and 2.49
[50]	0%, 10%, 20%, 30%, 40%, 50%, and 60%	3.8, 4.2, 5.1, 6.7, 6.3, 4.5, and 3.7

**Table 5:** Summary of flexural strength.

Reference	Iron Ore Tailing (IOT)	Flexure Strength (MPa)
[37]	0%, 20%, 40%, 60%, 80%, and 100%	7 Days
		14, 16, 14, 15, 15, and 15.5
		28 Days
		19, 18, 16, 17, 16.5, and 16
[36]	0%, 10%, 20%, 30%, 40%, and 50%	28 Days
		5.5, 5.4, 5.6, 5.5, 5.3, and 5.4
		56 Days
		5.8, 5.9, 6.1, 6.0, 5.9, and 5.8
[49]	0%, 10%, 20%, 30%, 40%, and 50%	3 Days
		6.86, 6.53, 5.13, 5.27, 5, and 5.4
		7 Days
		7.6, 6.8, 6.6, 5.5, 7.2, and 6.1
		28 Days
		10, 8.65, 7.5, 8.8, 9.2, and 7.4
		56 Days
		10.33, 8.67, 7.67, 8.93, 9.27, and 8
[55]	0%, 10%, 20%, 30%, and 40%	28 Days
		6.1, 6.2, 7.1, 7.0, and 7.8
[54]	0%, 6%, 12%, 18%, 24%, and 30%	14 Days
		1.3, 1.4, 1.5, 1.4, 1.3, and 1.2
		28 Days
		1.5, 1.6, 1.8, 1.7, 1.6, and 1.4
[57]	0%, 10%, 20%, 30%, and 40%	28 Days
		4.45, 4.52, 4.47, 4.79, and 4.41
[50]	0%, 10%, 20%, 30%, 40%, 50%, and 60%	5.2, 5.7, 6.5, 7.3, 7.2, 5.6, and 4.8

## Data Availability

Not applicable.
